# Differential expression of viral PAMP receptors mRNA in peripheral blood of patients with chronic hepatitis C infection

**DOI:** 10.1186/1471-2334-7-136

**Published:** 2007-11-19

**Authors:** Rafael Atencia, Francisco J Bustamante, Andrés Valdivieso, Arantza Arrieta, Marta Riñón, Alvaro Prada, Natalia Maruri

**Affiliations:** 1 Laboratorio de Inmunología, Hospital de Cruces, Barakaldo, Vizcaya, Spain; 2 Departamento de Gastroenterología y Hepatología, Hospital de Cruces, Barakaldo, Vizcaya, Spain; 3 Unidad de Cirugía Hepática. Hospital de Cruces, Barakaldo, Vizcaya, Spain

## Abstract

**Background:**

Pathogen-associated molecular patterns (PAMP) receptors play a key role in the early host response to viruses. In this work, we determined mRNA levels of two members of the Toll-like Receptors family, (TLR3 and TLR7) and the helicase RIG-I, all of three recognizing viral RNA products, in peripheral blood of healthy donors and hepatitis C virus (HCV) patients, to observe if their transcripts are altered in this disease.

**Methods:**

IFN-α, TLR3, TLR7 and RIG-I levels in peripheral blood from healthy controls (n = 18) and chronic HCV patients (n = 18) were quantified by real-time polymerase chain reaction.

**Results:**

Our results show that IFN-α, TLR3, TLR7 and RIG-I mRNA levels are significantly down-regulated in patients with chronic HCV infection when compared with healthy controls. We also found that the measured levels of TLR3 and TLR7, but not RIG-I, correlated significantly with those of IFN-α

**Conclusion:**

Monitoring the expression of RNA-sensing receptors like TLR3, TLR7 and RIG-I during the different clinical stages of infection could bring a new source of data about the prognosis of disease.

## Background

Virus infection initiates a series of cellular events that lead to the generation of an antiviral state both in the infected cell and the surrounding tissue [1]. Toll-like receptors (TLRs), an evolutionary conserved class of receptors found in plants, Drosophila and humans, play a critical role in the acquisition of this antiviral state. These receptors recognize pathogen-associated molecular patterns (PAMPs) and elicit antimicrobial immune responses. Until now, 10 different human toll-like receptors have been described and several from these recognize viral products [2,3]. Among the mammalian TLRs, three are related to recognition of RNA. TLR3 recognizes double-stranded dsRNA [4] of viral origin and is expressed preferentially in dendritic cells [5]. Once engaged, TLR3 triggers the activation of Interferon-regulatory factor 3 (IRF-3), a transcription factor playing a critical role in the induction of type I interferon and NF-κB through signaling processes that require the protein Toll-interleukin-1 receptor-domain-containing adaptor inducing IFN-β (TRIF) [6,7]. The type I IFN further upregulates TLR3 in an autocrine/paracrine manner, a phenomenon linked to its anti-viral gene defense action [8]. However, with regard to dsRNA, additional pattern-recognition receptors have been identified as candidates to initiate additional signalling pathways. One of these, retinoic-acid inducible gene-I (RIG-I), has recently been identified [9] and seems to be emerging as a key player in the induction of an interferon response by viruses. RIG-I encodes for a RNA-dependent helicase that is located cytoplasmically and is able to transmit downstream signals to activate NF-κB and IRF-3. The triggering of RIG-I could be induced from inside cell by replicating viruses. Moreover, RIG-I appears to have, like TLR3, a role in sensing HCV infection, thus forming an alternative pathway to establish an antiviral state [10].

TLRs 7 and 8 are close related phylogenetically and both are sensors for viral, single-stranded ssRNA [11,12]. Toll-like receptor 7 appears to be preferentially expressed by plasmacytoid dendritic cells and B lymphocytes whereas TLR8 is expressed at moderate levels in monocytes [5]. These TLRs also trigger IRF-7 mediated type I IFN production upon activation, but unlike TLR3, the induction of IFN by TLR7 and 8 is coupled to the adaptor protein MyD88 and not to TRIF [13].

Hepatitis C Virus (HCV) is a single-strand RNA virus that infects liver and lymphoid cells [14]. Currently, an estimated 3% of the world's population -more than 170 million people- is infected with HCV [3]. HCV causes acute and chronic hepatitis, and hepatocellular carcinoma [15], and chronic HCV infection is the most common cause of liver transplantation [16]. HCV is a single-stranded ss-RNA virus and therefore susceptible to detection by TLR7 and 8. Nevertheless, its genome also encodes regions of extensive secondary dsRNA structure that could be engaged by other PAMP receptors during infection [1]. Moreover, as a positive ssRNA virus, replication of HCV takes place through a minus-strand intermediate in a membrane-bounded compartment [17]. Therefore, the replicative machinery of HCV yields dsRNA intermediates that are likely exposed to the cell dsRNA-sensing receptors, such as TLR3 [18]. With these data in mind, the aim of this work was to determine the relative levels of TLR3, 7 and RIG-I mRNA expression in patients with and without chronic HCV infection and examine the potential of these TLRs as biomarkers for HCV infection.

## Methods

Patients with virologically and biochemically diagnosed chronic hepatitis C (n = 18) and a control group established with samples from healthy blood donors (n = 18) obtained from the Basque Transfusion Centre were used for this study. None of patients had received antiviral treatment before entry into the study, in order to avoid the possible effect of therapy on the expression of TLRs (such effect has been described by Vollmer *et al. *[19]). From the group of patients, whose clinical data are resumed in table 1, 8 had histologically or clinically confirmed cirrhosis (Child-Pugh A n = 2, Child-Pugh B n = 6). Routine laboratory methods were used to determine serum aspartate aminotransferase (AST), alanine aminotransferase (ALT) and viral load in HCV patients. All patients gave their written informed consent for the study. The study was approved by the hospital ethics committee (Comisión de Etica de la Investigación del Hospital de Cruces).

**Table 1:** Characteristics of HCV-infected patients included in this study

**Parameter**		**Value**
Gender (male/female)		9/9
Age		49,7 ± 17
Duration of disease	> 15 yr	7
	unknown	11
Viral Genotype	1b	10
	3a/4a	4
	unknown	4
Viral load (IU/ml)		1,96 × 10^6 ^± 0,80 × 10^6^
ALT/AST levels (IU/ml)		94 ± 24/74 ± 23

Data are expressed as mean ± standard error

Peripheral blood was collected in PAXGene blood RNA tubes (PreAnalytix GmbH, Switzerland). Then, total RNA was extracted using the PAXGene RNA purification kit (Qiagen GmbH, Germany) and purified with an intermediate, on-column, digestion with RNAse-free DNAse set (Qiagen). The quantity and purity of RNA obtained was assessed by measuring absorbance at 260 nm and the ratio A_260_/A_280 _in a UV-spectrophotometer (Bio-Rad). Only samples with an A260/A280 ratio from 1.9 to 2 were considered valid for RT-PCR.

500 ng of total RNA per sample was reverse transcribed using QuantiTect reverse transcription kit (Qiagen). Briefly, samples were incubated for 2 minutes at 42°C in a wipeout buffer to remove traces of genomic gDNA. Then, reverse transcriptase and RT primer mix were added and samples were incubated for 20 minutes at 42°C. A final incubation for 3 min at 95°C was performed to inactivate reverse transcriptase. In all cases, the presence of gDNA was excluded by performing the adequate control reactions without reverse transcriptase.

The PCR for TLRs mRNA quantification was performed with the LightCycler FastStart DNA SYBR-Green I kit (Roche Applied Science, RAS, Mannheim, Germany) according to the protocol provided in the parameter-specific Kits. Specific primer sets [5] optimized for the LightCycler (RAS) were developed and purchased from SEARCH-LC GmbH, Heidelberg [30]. Human porphobilinogen deaminase (PBGD) was used as housekeeping gene for internal control. To control for specificity of the amplification products, a melting curve analysis was performed. No amplification of unspecific products was observed.

For quantification and statistical analysis, target genes mRNA expression was normalized to the expressed housekeeping gene PBGD using Relative Expression Software Tool 2005 [20], which uses the pair-wise fixed reallocation randomization test as statistical model. This approach overcomes the problems that make very difficult to perform traditional statistical analysis, such as the absence of standard deviation in ratio distributions. Correlation analysis was performed using non parametric Spearman-Kendall test, and a value of p < 0.05 was considered as statistically significant.

## Results and discussion

**Figure 1:**
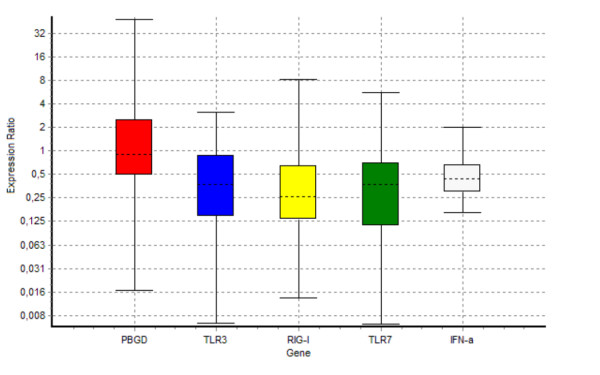
**Relative expression of IFN-α, TLR3, RIG-I and TLR7 obtained after analysis of RT-PCR data with REST**. Boxes represent the interquartile range, or the middle 50% of observations. The dotted line represents the median gene expression. Whiskers represent the minimum and maximum observations.

In this preliminary work, we have used quantitative RT-PCR to evaluate the expression of PAMP receptors related with the recognition of viral RNA in peripheral blood of healthy controls and HCV-infected patients. The mRNA relative expression values for IFN-α, TLR3, TLR7 and RIG-I are depicted in table 2 and illustrated in figure 1. It is important to note here that the whisker-box plots that the REST software draws in its reports (reproduced here in figure 1) are based upon permuted expression data rather than the raw CP values obtained from the RT-PCR step, because, as we described above, REST 2005^® ^uses randomization techniques. The analysis of the RT-PCR data through REST 2005^® ^software shows that levels of the two dsRNA sensing receptors TLR3 and RIG-I are significantly lower in patients with chronic hepatitis C when compared with healthy individuals (p = 0.008 and p = 0.002 respectively). With regard to TLR7 and IFN-α, there is also a significant lower expression in HCV patients (p = 0.004 and p = 0.015 respectively).

**Table 2:** Statistical analysis summary of results obtained after processing RT-PCR data through REST (Relative Expression Software Tool)

Gene	Reaction Efficiency	Expression	Std. Error	95% C.I.	P(H1)	Result
PBGD	0,948	1,000	0,356 – 4,411	0,026 – 11,015	1,000	
TLR3	1,0	0,327	0,092 – 1,042	0,019 – 2,498	0,008	DOWN
RIG-I	1,0	0,295	0,094 – 0,898	0,031 – 4,221	0,002	DOWN
TLR7	1,0	0,289	0,074 – 0,953	0,015 – 2,383	0,004	DOWN
IFN-α	0,958	0,584	0,311 – 1,039	0,221 – 2,302	0,015	DOWN

The parameters used for analysis were a normalization factor of 1.27 and 50000 iterations of randomization test.

**Figure 2:**
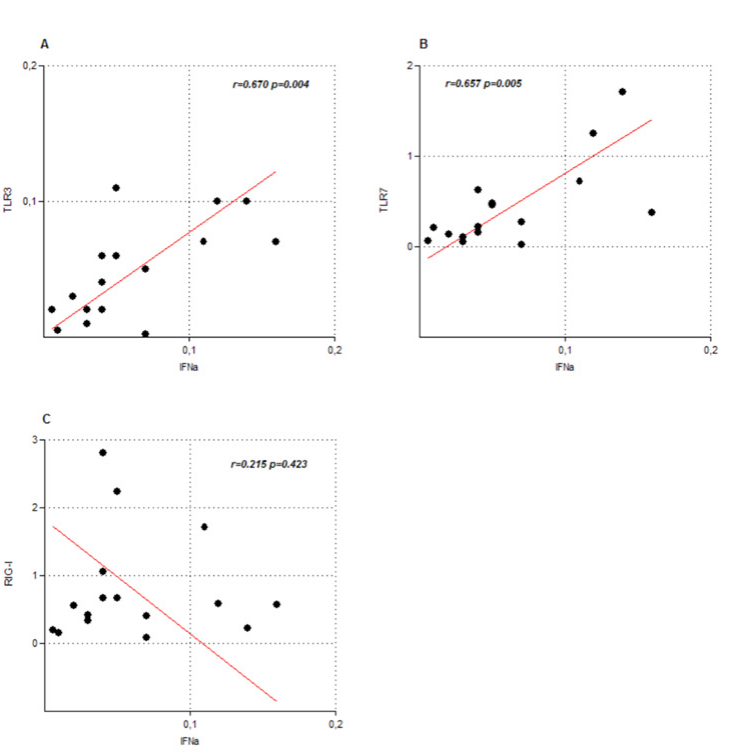
**Correlation plots between the mRNA relative levels of IFN-α and PAMP receptors**. Each plot shows the correlation between the expression levels of IFN-α against those of TLR3 (A), TLR7 (B) or RIG-I (C)

The statistical regression analysis of the quantitative RT-PCR data and clinical data showed no correlation between the expression levels of the studied receptors and other factors as viral load, AST/ALT levels or cirrhosis. However, we found that the measured levels of TLR3 and TLR7, but not RIG-I, correlated significantly with those of IFN-α(r = 0.670, p = 0.004 and r = 0.657, p = 0.005 respectively. Figure 2).

The RNA-sensing Toll-like receptors have been related with the recognition and interferon-mediated response against viruses [21-24] showing that they play a key role in the innate response against viral infections. With regard to HCV, it has been recently demonstrated that the viral NS3/4A protease cleaves the TLR3 adaptor, TRIF [25], and also targets CARDIF, an adaptor protein that links RIG-I to the start of antiviral response [26] as a mechanism of immune evasion. However, most of these reports have been developed on in vitro or animal experimental systems. Here we show preliminary data of association between the expression of TLR3, TLR7, RIG-I and HCV infection in a clinical context. These receptors are down-regulated in patients with chronic HCV infection, and the expression levels of TLR3 and TLR7 (but not RIG-I) correlate strongly with the expression level of IFN-α, suggesting that one of the mechanisms leading to the chronicity of infection could be the acquisition of an "exhausted" state of this antiviral machinery at the late stages of infection. Moreover, this down-regulation appears to be related with the infection, because the same analysis performed on patients with liver cirrhosis not related with viral infections (mainly of alcoholic origin) did not show significant differences when compared with healthy controls (our unpublished results). With regard to the lack of correlation between the RIG-I and IFN-α expression levels, these results could be explained by the fact that plasmacytoid dendritic cells (the main producers of IFN-α at the innate immune system) use the TLR system rather than RIG-I for viral detection [27].

Our results are in agree with those of Taylor et al [28], in which a reduced level of expression of TLR7 is observed in HCV patients with poor response to interferon therapy. Nevertheless, our results contrast with those recently published by Dolganiuc et al. [29], who show a wide up-regulation of almost every TLR (including TLR3 and TLR7) in monocytes and lymphocytes of patients with chronic HCV infection. However, this noticeable contradiction between these two sets of results must be analysed with care. First of all, the two sets of results are not directly object of comparison, for the reason that different methodological approaches were used (cellular separation vs. total blood, differences in housekeeping genes, different algorithms and statistical tools applied to results from RT-PCR assays...). Despite the benefits of relative quantification using RT-PCR in terms of sensibility and accuracy, a standardised methodology for normalisation and analysis of results remains to be widely accepted and, meanwhile, every result must be observed cautiously. However, in this case, we regard as a more significant issue to explain this opposite results that the group of patients included in the study by Dolganiuc *et al. *showed clinical parameters suggesting a less advanced stage of disease that the ones included in this work (i.e., none of the patients included in that study had cirrhosis at the time of inclusion as soon as the average viral load was almost a log decade higher). Thereby, those differences in clinical stage could explain the discrepancy between the two set of results and hint for a kinetic of these receptors related to the course of infection. Thus, the monitoring of these TLR levels during the course of infection could bring a new source of data about the prognosis of disease by using non-invasive techniques.

Nevertheless, the data presented here should be observed only as part of a more complex picture. As it has been written above, the HCV has molecular strategies to evade signalling at least by TLR3 and RIG-I, thus leading to a defective IFN response. Therefore, as our preliminary results show, it could be of interest a monitoring of production of interferon α and β simultaneously to the PAMP receptors expression and try to establish a correlation pattern between PAMP receptor levels and IFN levels, because these data could allow detecting those evasive manoeuvres by HCV in its road towards chronicity.

## Conclusion

The data presented here should be considered as a preliminary report from a work in progress that we are currently developing at our laboratory, but they point to the fact that measuring the expression of RNA-sensing receptors like TLR3, TLR7 and RIG-I could provide a new set of molecular markers for the prognosis of the HCV infection. Nevertheless, further research covering other stages of disease (like the acute resolving phase of infection) will be needed to confirm their real value.

## Competing interests

The author(s) declare that they have no competing interests.

## Authors' contributions

RA, conceived the study, participated in its design, carried out the quantitative RT-PCR studies and drafted the manuscript. FJB and AV participated in the design of the study, provided the patients samples and the biochemical data. MR and AP performed the RNA purification. AA performed the statistical analysis. NM participated in the design and coordination of the study and helped to draft the manuscript. All authors read and approved the final manuscript.

## Pre-publication history

The pre-publication history for this paper can be accessed here:



## References

[B1] Gale M, Foy EM (2005). Evasion of intracellular host defence by hepatitis C virus. Nature.

[B2] Takeda K, Kaisho T, Akira S (2003). Toll-like receptors. Ann Rev Immunol.

[B3] Kawai T, Akira S (2005). Pathogen recognition with Toll-like receptors. Curr Opin Immunol.

[B4] Alexopoulou L, Holt AC, Medzhitov R, Flavell RA (2001). Recognition of double-stranded RNA and activation of NF-κB by Toll-like receptor 3. Nature.

[B5] Hornung V, Rothenfusser S, Britsch S, Krug A, Jahrsdorfer B, Giese T, Endres S, Hartmann G (2002). Quantitative expression of Toll-like receptor 1–10 mRNA in cellular subsets of human peripheral blood mononuclear cells and sensitivity to CpG oligodeoxynucleotides. J Immunol.

[B6] Schultz O, Diebold SS, Chen M, Naslund TI, Nolte MA, Alexopoulou L, Azuma YT, Flavell RA, Liljestrom P, Reis e, Sousa C (2005). Toll-like receptor 3 promotes cross-priming to virus-infected cells. Nature.

[B7] Yamamoto M, Sato S, Mori K, Oshino K, Takeuchi O, takeda K, Akira S (2002). Cutting edge: a novel Toll/IL-1 receptor domain adapter that preferentially activates the IFN-β promoter in the Toll-like receptor signalling. J Immunol.

[B8] Doyle SE, O'Connell R, Vaidya SA, Chow EK, Yee K, Cheng G (2003). Toll-like receptor 3 mediates a more potent antiviral response than Toll-like receptor 4. J Immunol.

[B9] Yoneyama M, Kikuchi M, Natsukawa T, Shinobu N, Imaizumi T, Miyagishi M, Taira K, Akira S, Fujita T (2004). The RNA helicase RIG-I has an essential function in double-stranded RNA-induced innate antiviral responses. Nat Immunol.

[B10] Sumpter R, Loo YM, Foy E, Li K, Yoneyama M, Fujita T, Lemon SM, Gale M (2005). Regulating Intracellular Antiviral Defense and Permissiveness to Hepatitis C Virus RNA Replication through a Cellular RNA Helicase, RIG-I. J Virol.

[B11] Diebold S, Kaisho T, Hemmi H, Akira S, Reis e Sousa C (2004). Innate antiviral responses by means of TLR7-mediated recognition of single-stranded RNA. Science.

[B12] Heil F, Hemmi H, Hochrein H, Ampenberger F, Kirschning C, Akira S, Lipford G, Wagner H, Bauer S (2004). Species-specific recognition of single-stranded RNA via Toll-like receptor 7 and 8. Science.

[B13] Akira S, Takeda K (2004). Toll-like receptor signalling. Nat Rev Immunol.

[B14] Lindenbach BD, Rice CM, Knipe DM, Howley PM Fields Virology.

[B15] Alter HJ, Seeff LB (2000). Recovery, persistence, and sequelae in hepatitis C virus infection: a perspective on long-term outcome. Semin Liver Dis.

[B16] Bizollon T, Ducerf C, Trepo C (1999). Hepatitis C virus recurrence after liver transplantation. Gut.

[B17] Moradpour D, Brass V, Bieck E, Friebe P, Gosert R, Blum HE, Bartenschlager R, Penin F, Lohmann V (2004). Membrane association of the RNA-dependent RNA polymerase is essential for hepatitis C virus RNA replication. J Virol.

[B18] Samuel CE (2001). Antiviral actions of interferons. Clin Microbiol Rev.

[B19] Vollmer J, Rankin R, Hartmann H, Jurk M, Samulowitz U, Wader T, Janosch A, Schetter C, Krieg AM (2004). Immunopharmacology of CpG oligodeoxynucleotides and rivabirin. Antimicrob Agents Chemother.

[B20] Pfaffl MW, Horgan GW, Dempfle L (2002). Relative expression software tool (REST) for group-wise comparison and statistical analysis of relative expression results in real-time PCR. Nucleic Acids Res.

[B21] Guillot L, Le Goffic R, Bloch S, Escriou N, Akira S, Chignard M, Si-Tahar M (2005). Involvement of Toll-like receptor 3 in the immune response of lung epithelial cells to double-stranded RNA and influenza A virus. J Biol Chem.

[B22] Hewson CA, Jardine A, Edwards MR, Laza-Stanca V, Johnston SL (2005). Toll-like receptor 3 is induced by and mediates antiviral activity against rhinovirus infection of human bronchial epithelial cells. J Virol.

[B23] Rudd BD, Burstein E, Duckett CS, Xiaoxia L, Lucaks NW (2005). Differential role for TLR3 in respiratory syncytial virus-induced chemokine expression. J Virol.

[B24] Sanghavi SK, Reinhart TA (2005). Increased expression of TLR3 in lymph nodes during simian immunodeficiency virus infection: implications for inflammation and immunodeficiency. J Immunol.

[B25] Li K, Foy E, Ferreon JC, Nakamura M, Ferreon AC, Ikeda M, Ray SC, Gale M, Lemmon SM (2005). Immune evasión by hepatitis C virus NS3/4A protease-mediated cleavage of the Toll-like receptor 3 adaptor protein TRIF. Proc Natl Acad Sci USA.

[B26] Meylan E, Curran J, Hofmann K, Moradpour D, Blinder M, Bartenschlager R, Tschopp J (2005). Cardif is an adaptor protein in the RIG-I antiviral pathway and is targeted by hepatitis C virus. Nature.

[B27] Kato H, Sato S, Yoneyama M, Yamamoto M, Uematsu S, Matsui K, Tsujimura T, Takeda K, Fujita T, Takeuchi O, Akira S (2005). Cell type-specific involvement of RIG-I in antiviral response. Immunity.

[B28] Taylor MW, Tsukahara T, Brodsky L, Schaley J, Sanda C, Stephens MJ, McClintick JN, Edenberg HJ, Li L, Tavis JE, Howell C, Belle SH (2007). Changes in gene expression during peginterferon and ribavirin therapy of chronic hepatitis C distinguish responders from non responders to antiviral therapy. J Virol.

[B29] Dolganiuc A, Garcia C, Kodys K, Szabo G (2006). Distinct toll-like receptor expression in monocytes and T cells in chronic HCV infection. World J Gastroenterol.

[B30] Search-LC LightCycler Reagents for Quantitative PCR. http://www.search-lc.com.

